# Water-Soluble Intracellular Polysaccharides (IPSW-2 to 4) from *Phellinus igniarius* Mycelia: Fractionation, Structural Elucidation, and Antioxidant Activity

**DOI:** 10.3390/foods13223581

**Published:** 2024-11-09

**Authors:** Isaac Duah Boateng, Xiaoming Yang

**Affiliations:** 1 School of Food & Biological Engineering, Jiangsu University, Zhenjiang 212013, China; boatengisaacduah@gmail.com; 2 Certified Group, 199 W Rhapsody Dr, San Antonio, TX 78216, USA

**Keywords:** *Phellinus igniarius* mycelia, polysaccharides, waste valorization, structural analysis, NMR, GC-MS, antioxidant activity

## Abstract

*Phellinus igniarius* is a medicinal fungus. Nonetheless, research on its water-soluble intracellular polysaccharides (IPSW-2 to 4) fractionation, structural elucidation, and antioxidant activity is limited. In this study, water-soluble intracellular polysaccharides (IPSW-2 to 4) were extracted and fractionated from *P. igniarius* mycelia, and their antioxidant and structural properties were assessed using GC-FID, GC-MS, FTIR, and NMR spectroscopy (^1^H and ^13^C). In the water-eluted *P. igniarius* polysaccharide fractions (IPS30W, IPS60W, and IPS80W) of anion-exchange chromatography, the polysaccharide content was 79.05%, 68.25%, and 62.06%, with higher yields of 25.07%, 21.38%, and 20.34%, respectively. In contrast, the salt (NaCl) elution fractions (IPS30S1, IPS60S1, IPS60S2, and IPS80S1) of anion-exchange chromatography had lower polysaccharide content and yield. Hence, water elution fractions (IPS30W, IPS60W, and IPS80W) were selected for further purification. After repeated purification using size-exclusion chromatography, IPSW-2 to 4 were obtained with a yield of 8% to 15.83%. The IPSW-2 to IPSW-4 structures were elucidated, and they showed no triple helical conformation. Based on periodate oxidation, Smith degradation, methylation analysis, and ^1^H and ^13^C NMR spectroscopy, the primary structures of IPSW-2, IPSW-3, and IPSW-4 were all glucan, with the main chain consisting of (1→6)-α-D-Glc*p*, (1→3,4)-α-D-Glc*p*, and (1→3, 6)-α-D-Glc*p*, with α-D-Glc*p* as a side chain. Finally, antioxidant analysis showed that IPS30W, IPS60W, and IPS80W were all more capable of scavenging superoxide anions than the polysaccharides of Phyllostachys (13.8%) and floribunda (15.1%) at the same concentration (0.40 mg/mL). This will serve as a guide for the development of functional foods.

## 1. Introduction

Edible and medicinal fungi containing bioactive compounds have traditionally been used to cure or prevent various ailments. As one of the high-content components in mushrooms, fungal polysaccharides have been extensively studied [1,2,3], and some significant improvements have been made in recent decades [4]. Polysaccharides from fungi have been shown to have a wide range of pharmacological properties, including immunological modulation, anticancer, anti-inflammation, and antioxidant activity [5]. Some polysaccharide fractions derived from fungi have been researched as medicines and functional foods for various applications [6,7].

*Phellinus igniarius* is a precious medicinal fungus and is mainly parasitic on poplar, willow, oak, pine, and other trees. Occasionally, it also grows on other angiosperms and is widely distributed [8,9]. In China, the producing areas are concentrated in the Changbai Mountain forest area in the northeast and the eastern part of Heilongjiang Province, between the Wusuli River and Xingkai Lake, at the junction of Shaanxi and Gansu in the northwest region; it also grows in forest reserves in Japan, Australia, South Korea, Russia, and other countries. Moreover, *P. igniarius* was documented in the “Compendium of Materia Medica” written more than 2000 years ago [10]. Studies have shown that *Phellinus igniarius*’s main chemical composition includes polysaccharides, triterpenes, flavones, amino acids, aromatic acids, etc., and polysaccharides account for the largest proportion [11]. It also has anti-tumor, anti-aging, and anti-inflammation properties; improves immunity; and lowers blood sugar [8,12,13]. Moreover, studies have shown that the anti-tumor effects of *P. igniarius* are better than those of *Ganoderma lucidum* and medicinal fungi such as Brazilian mushrooms [8], and they have received widespread attention [6,7,14,15].

The extraction and separation of polysaccharides is a critical step in the research and application of *P. igniarius*. During this process, it is necessary to prevent structural changes and the loss of biological activity [14,15]. Water extraction and ethanol precipitation are the most commonly used methods for the extraction of polysaccharides, and the hot-water extraction process is also accompanied by the dissolution of water-soluble impurities; for example, proteins that accompany polysaccharides precipitate together in crude polysaccharides [16]. The main methods for separating and purifying polysaccharides are chromatography, ultrafiltration, and non-solvent precipitation [17]. Research on the polysaccharides of *P. igniarius* has mainly focused on optimizing the extraction process [15]. Wu et al. [18] separated a polysaccharide (PIP1) from *P. igniarius*’s mycelium; the main chain was made up of glucose (1→3) and mannose (1→4), and the side chain was made of glucose (1→3) and galactose (1→6). Gao et al. [19] optimized the sonoprocessing of *Phellinus linteus* mycelium polysaccharides, and Yuan et al. [5] isolated a strain of *P. igniarius* from the fruiting body and found that the structure was a linear repeating backbone made of galactopyranose, glucopyranose (Glc*p*), and mannopyranose connected by α-(1→3), α-(1→4), and α-(1→6) linkages and single α-terminal-D-Glc*p* as side chains 6-*O*-linked to the main chain and that it exhibited a triple helical structure. Suabjakyong et al. [20] separated polysaccharides from *P. igniarius*’s mycelium, and the main chain was made up of glycosidic linkages 1→3, 1→6, or 1→3,6 from glucose and a side chain of α-D-Man*p*-(1→. In recent research by Zhang et al. [21], *P. igniarius* SH-1 was fermented (using strain OR016144), and PIPS-2 was isolated and purified. Structural analysis showed that the primary chain of PIPS-2 entailed →2)-α-D-Man*p*-(1→3)-β-D-Galf-(1→, and the side chains involved α-D-Man*p*-(1→6)-α-D-Man*p*-(1→, α-D-Manp-(1→3)-α-D-Man*p*-(1→, and α-D-Man*p*-(1→.

Our previous study effectively cultivated *P. igniarius* mycelium strain No. 5.95 via liquid fermentation [14], and the results from anion-exchange and size-exclusion (two-stage) chromatography showed that IPSW-1, IPSW-2, IPSW-3, and IPSW-4 were homogenous polysaccharides with molecular weights (Mw) of 34.1 kDa, 17.7 kDa, 15.1 kDa, and 21.7 kDa, respectively. However, research on the structural elucidation of IPSW-2 to IPSW-4 using GC-FID, GC-MS, and nuclear magnetic resonance (NMR) is limited. Moreover, research on comprehensive fractionation (ethanol precipitation and water- and salt-eluted (0 to 0.4 mol/L)), purification, structural elucidation, and antioxidant activity of IPS from *P. igniarius* mycelia is limited. Therefore, in this work, water-soluble polysaccharides (IPSW-2 to 4) were extracted, fractionated, and purified from *P. igniarius* fermentation mycelia. A comprehensive analysis was performed on the compositions of various fractionated fractions from the separation process. Moreover, the structural characteristics were investigated by Smith degradation using periodate oxidation reactions and GC-FID; methylation using GC-MS and FTIR; and NMR spectroscopy, including ^1^H and ^13^C. The positions of the bonds were deduced by KMnO_4_ and NaIO_4_ oxidative decomposition. Finally, the antioxidant capacities of IPS were assessed using various assays.

## 2. Materials and Methods

### 2.1. Materials and Reagents

*P. igniarius* No. 5.95 was purchased from the China General Microbiological Culture Collection Center (CGMCC, Beijing, China) and was submerged in a culture to provide *P. igniarius* mycelia [14]. Nitro blue tetrazolium (NBT) was purchased from Nanjing Shengxing Biotechnology Co., Ltd. (Nanjing, China), and 2,2-diphenyl-1-picryl-hydrazyl (DPPH) was purchased from Sigma-Aldrich (Merck Millipore, Darmstadt, Germany). Phenazine dimethyl sulfate (PMS) was purchased from Shanghai Yuanju Biotechnology Co., Ltd. (Shanghai, China). Other chemicals and reagents were purchased from the Shanghai reagent company. All reagents were of analytical grade or the highest grade available and were used without further purification.

### 2.2. Extraction, Fractionation, and Purification of Polysaccharides

**Figure 1:**
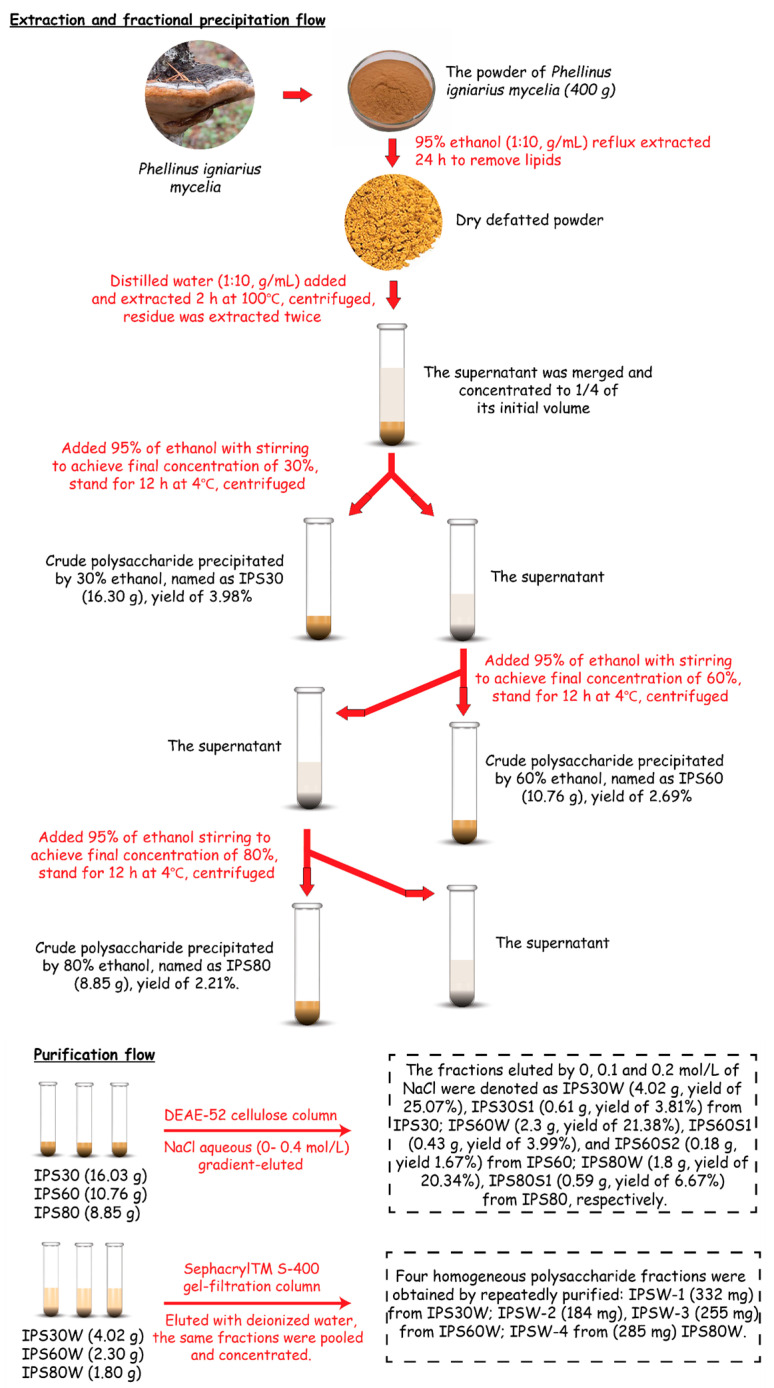
Flow diagram of the extraction, fractionation, and purification of water-soluble intracellular polysaccharides from *P. igniarius* mycelia.

The water-soluble intracellular polysaccharides (IPSs) of *P. igniarius* in this study were extracted and purified [22] as shown in Figure 1. The extraction yield was calculated [23].

### 2.3. Molecular Characterization Analysis

#### 2.3.1. Determination of Polysaccharide and Protein Content

Total carbohydrate was assessed using the phenol–sulfuric acid protocol [24] with minor modifications. The polysaccharide solution was mixed with phenol solution in water (80% *v*/*v*), and then concentrated H_2_SO_4_ (1.2 mL) was added immediately, followed by vortexing. The mixture was then allowed to stand at 25 °C for 30 min. A spectrophotometer (UV-VIS) was employed to measure the absorbance of the solution at 490 nm. A calibration curve was prepared using glucose, and dH_2_O was used instead of the sample solution as a blank. The protein was evaluated by applying the Bradford method [25] with bovine serum protein (BSA) as the standard.

#### 2.3.2. FT-IR Spectroscopy

The Li et al. [26] protocol was used for the FT-IR spectrum of IPSW-2 to IPSW-4 using a Nexus 470 FT-IR spectrometer (Thermo Fisher Co, Austin, TX, USA) at 400–4000 cm^−1^ with KBr [26].

#### 2.3.3. Periodate Oxidation

The periodate oxidation reactions of IPSW-2 to IPSW-4 were performed according to the procedure detailed by Boateng et al. [22].

#### 2.3.4. Smith Degradation Using Gas Chromatography Flame Ionization Detector (GC-FID)

IPSW-2 to 4 (20 mg) were placed into a test tube, and 20 mL of NaIO_4_ solution (15 mmol/L) was added, sealed, and shaken at 4 °C in the dark for 168 h until the absorbance at 223 nm became stable, after which 2.0 mL of ethylene glycol was added and stirred for 30 min to reduce the remaining NaIO_4_. The product was dialyzed against tap-flowing and distilled water for 48 h to remove many small-molecule reactants and products. The dialysate was concentrated using a rotary evaporator (50 °C) to ~5 mL, and NaBH_4_ (40 mg) was added and stirred in the dark to reduce polysaccharide aldehyde [27].

The pH was adjusted to 5.5 with acetic acid (0.1 mol/L) to decompose the remaining borohydride compounds and dialyzed again as described above. The dialysates were concentrated and freeze-dried to obtain polysaccharide, which was dissolved in 5 mL of H_2_SO_4_ (2 mol/L) and hydrolyzed at 100 °C for 8 h. This was followed by acid removal by neutralization with BaCO_3_, and then the samples were centrifuged (4000 rpm for 5 min). The supernatant was evaporated to dryness, and residuals were treated with hydroxylamine hydrochloride (10 mg) and 1.0 mL of pyridine at 90 °C for 30 min. The resulting solution was cooled to room temperature and then heated at 90 °C for 30 min using 1.0 mL of acetic anhydride to obtain the final products.

The final products were analyzed by GC (Agilent 7890 A, Santa Canta, CA, USA) with an HP-5 column (30 m × 320 × 0.25 μm) and a flame ionization detector (FID) at a column flow rate of 1 mL/min. Chromatographic programming was performed using the protocol by Li et al. [14]. The glycerol and erythritol were treated with hydroxylamine hydrochloride, pyridine, and acetic anhydride to obtain their derivatization products and analyzed by GC.

#### 2.3.5. Methylation Analysis Using GC-MS

A methylation analysis of IPSW-2 to IPSW-4 was conducted using Boateng et al.’s [22] method. The dried hydrolysates (samples completely hydrolyzed by acid (formic acid + trifluoroacetic acid)) were dissolved in 2 mL of H_2_O and reduced with NaBH_4_ (25 mg) for 2 h at 25 °C. The excess NaBH_4_ was decomposed by adding acetic acid and evaporated to dryness. The formed borate acid was removed by distillation with 5 mL of methanol, and a drop of acetic acid was added and evaporated to dryness using a rotary evaporator. This process was repeated 5 times (the last 2 times without adding acetic acid). Next, the dried reduced products were acetylated with 2 mL acetic anhydride at 100 °C for 1 h in a sealed test tube, and decompression evaporation was applied to dry the product. After evaporating the products 3 times with 3 mL of toluene, the resulting methylated derivatives were finally redissolved in chloroform and subjected to GC-MS analysis.

A Trace GC Ultra/ITQ1100 GC-MS (Thermo Scientific, Austin, TX, USA) fitted with an HP-5 capillary column (30 m × 0.32 mm × 0.25 μm) was used. Chromatographic programming was performed using the protocol by Boateng et al. [22].

#### 2.3.6. NMR Spectroscopy

The IPSW-2 to IPSW-4 were dissolved in deuterium oxide (D_2_O, 99.99%, 60 mg/mL) [28,29]. The NMR spectra were recorded using a Bruker AVANCE III 400 MHz spectrometer (Bruker, Rheinstetten, Germany) operating at 400 MHz for ^1^H analysis and at 125 MHz for ^13^C analysis at 25 °C [22]. The chemical shifts of the polysaccharide were expressed in δ (ppm) [30].

### 2.4. Antioxidant Activity Assays of IPS30, IPS60, and IPS80

#### 2.4.1. Superoxide Radical Scavenging Capacity Assay (SRSCA)

The method of Gharib et al. [31] with minor modification was used for SRSCA. The sample solution (1.5 mL) was mixed with 0.5 mL of nitroblue tetrazolium (300 µmol/L) and 0.5 mL NADH (468 µmol/L). After thorough mixing, the 0.5 mL phenazine methosulfate (PMS, 0 µmol/L) was added and incubated (5 min at 25 °C). The absorbance (A_sample_) was measured at 560 nm. Tri-HCl buffer replaced the sample as a blank control. The SRSCA was calculated as shown below:

$$\mathrm{Superoxide}\;\mathrm{radical}\;\mathrm{scavenging}\;\mathrm{capacity}=\frac{A_{\mathrm{control}}-A_{\mathrm{sample}}}{A_{\mathrm{control}}}\times 100\%$$

#### 2.4.2. Hydroxyl Radical Scavenging Capacity (HRSA)

The HRSA was assessed as described by Peng et al. [32] with minor modifications. First, 1.0 mL of 1,10-phenanthroline ethanol solution (0.75 mmol/L) was mixed well with 2 mL of PBS (pH 7.4) and 1.0 mL distilled water. Then 1.0 mL FeSO_4_ (0.75 mM) and 1.0 mL H_2_O_2_ (0.01%) were added. The final mixture was incubated (37 °C for 60 min). Distilled water (1.0 mL) instead of H_2_O_2_ was used as the control, and 1.0 mL of sample instead of distilled water was used as the sample test. The absorbance values of the control (A_control_), the blank (A_blank_), and the sample solutions (A_sample_) were recorded at 536 nm. The HRSA was computed using the equation below:

$$\mathrm{Hydroxyl}\;\mathrm{radical}\;\mathrm{scavenging}\;\mathrm{capacity}=\frac{A_{\mathrm{sample}}-A_{\mathrm{blank}}}{A_{\mathrm{control}}-A_{\mathrm{blank}}}\times 100\%$$

#### 2.4.3. Reducing Power

The reducing power was assessed as described by Zhou et al. [33] with minor modifications. The sample solution (1.0 mL) was mixed with 2.5 mL PBS (0.2 mol/L, pH 6.6) and 2.5 mL K_3_Fe(CN)_6_ (1%, *w*/*v*) and incubated (20 min at 50 °C). The reaction was halted by adding trichloroacetic acid (2.5 mL, 10%, *w*/*v*). The mixture solution was centrifuged (3000 rpm for 10 min), and supernatant (2.5 mL) was mixed with 0.5 mL of FeCl_3_ (0.1%, *w*/*v*), and the absorbance was measured at 700 nm.

#### 2.4.4. 2,2-diphenyl-1-picryl-hydrazyl Scavenging Capability (DPPH-SC)

The DPPH-SC of the samples was determined based on the protocol by Zhou et al. [33] with minor modifications. A sample solution (2 mL) of different concentrations was mixed with 2 mL of DPPH (0.04 mg/mL) ethanol solution, incubated for 30 min in darkness, and centrifuged (10 min at 3000 rpm). The absorbance of the sample solution (A_sample_) was measured at 517 nm. Then, 2.0 mL of ethanol instead of DPPH was used as the control. The DPPH-SC was calculated using the following equation:

$$\mathrm{DPPH}\;\mathrm{radical}\;\mathrm{scavenging}\;\mathrm{capability}\;=(1-\frac{A_{\mathrm{sample}}-A_{\mathrm{ethanol}}}{A_{\mathrm{DPPH}}})\times 100\%$$

### 2.5. Statistical Analysis

The experiment was conducted in triplicate. The results were presented as mean ± standard deviation. One-way analysis of variance (ANOVA) was applied to the results using Minitab Version 2021 (Minitab Inc., State College, PA, USA), and the Tukey test was performed with *p* < 0.05, signifying that the difference was statistically significant. Origin Pro 2021 (OriginLab^®^, Northampton, MA, USA) was used for the graphical representation.

## 3. Results and Discussion

### 3.1. Extraction, Purification, and Composition of Polysaccharides

The extraction and separation of polysaccharides is a key step in the research and application of *Phellinus igniarius* mycelia, and it is necessary to prevent structural changes and loss of biological activity during this process [34,35]. Hot water extraction and ethanol precipitation are the most commonly used methods for preparing polysaccharides, and the hot water extraction process is also accompanied by the dissolution of water-soluble impurities [16], which precipitate together with the polysaccharides in crude polysaccharides. The use of ethanol stepwise precipitation can reduce the workload of subsequent separation processes. The volume fraction of ethanol used for precipitation not only directly affects the yield of polysaccharides but also affects the properties of the resulting polysaccharides. Zhou et al. [36] reported on intracellular polysaccharides of *P. igniarius* mycelia precipitated by ethanol at different concentrations, and their findings showed that the physicochemical properties and antioxidant activity are different. Fractionation of polysaccharides is mainly performed by chromatography, ultrafiltration, and gradient non-solvent precipitation. Chromatography and ultrafiltration use columns and membranes to fractionate polysaccharides, respectively [17]. Among them, chromatography is the most widely used method. Generally, anion-exchange chromatography is used for preliminary fractionation, and gel chromatography is used for refining. DEAE-cellulose and Sephacryl^TM^ S-400 are typical anion-exchange chromatographic and gel chromatographic packing [34]. The *Phellinus igniarius* mycelia were extracted by hot water. After the extract was concentrated, 30%, 60%, and 80% ethanol gradients precipitation was used step by step. The crude polysaccharides were fractionated by an ion exchange column and a gel column.

**Figure 2:**
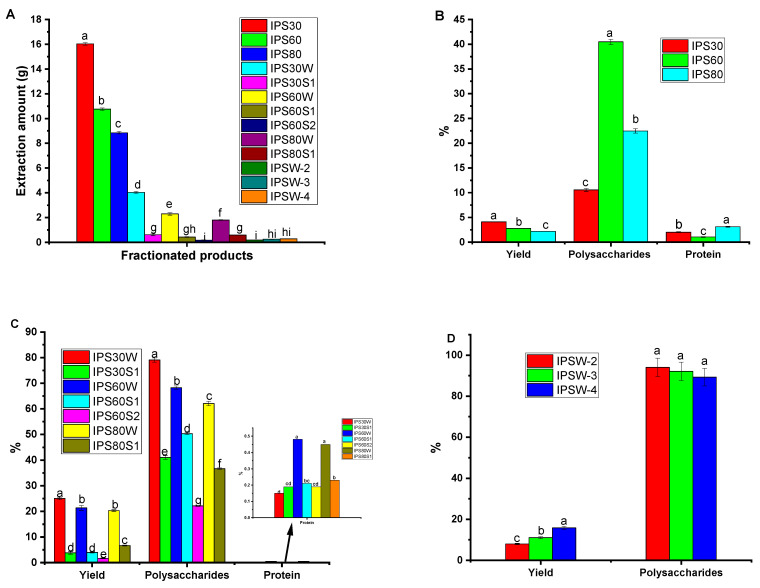
(**A**) Amounts of fractionated products. (**B**–**D**) The yield and content of carbohydrate and protein of (**B**) crude polysaccharides, (**C**) polysaccharides by DEAE-52 cellulose column chromatography, (**D**) polysaccharides by SephacrylTM S-400 gel-filtration column chromatography. Note: Different letters within groups indicate significant differences in the same column, *p* < 0.05. For (**C**), the yield (%) calculation is based on dry ethanol fractional polysaccharides IPS30, IPS60, and IPS80, respectively. For (**D**), the yield (%) calculation is based on dry separation products by DEAE-52 cellulose column chromatography. IPSW-1 is from IPS30W, IPSW-2 and IPSW-3 from IPS60W, and IPSW-4 from IPS80.

The yield, polysaccharides, and protein contents of crude polysaccharides and their various separated fractions are presented in Figure 2A–D. Figure 2A,B show the crude polysaccharides yield, polysaccharides, and protein contents (IPS30 to IPS80 extracted from *P. igniarius* mycelia using hot-water extraction and 30%, 60%, and 80% ethanol gradient precipitation). IPS30 had the highest amount of fractionated products (Figure 2A) with a significant difference (*p* < 0.05). This shows that the extraction yield of *P. igniarius* was greatly affected by the extraction solvents and their concentration, and this agrees with the findings of Dou et al. [37]. In Figure 2B, the polysaccharides of IPS30, IPS60, and IPS80 were 10.56%, 40.48%, and 22.48%, respectively (*p* < 0.05), with a small amount of protein (1.05–3.12%). Furthermore, IPS60 had the highest percentages of polysaccharide and protein (*p* < 0.05) compared with IPS30 and IPS80.

DEAE-52 cellulose resin has a strong binding capacity under the action of charge [38], and various fractions of polysaccharide elute out of the column at different solvents and concentrations. The polysaccharide was purified using a DEAE-52 cellulose column, and the percentage was calculated as shown in Figure 2C. It was observed that in the water elution fractions (IPS30W, IPS60W, and IPS80W), the polysaccharides content increased to 79.05%, 68.25%, and 62.06%, with higher yields of 25.07%, 21.38%, and 20.34% (calculated according to IPS30, IPS60, and IPS80), respectively (Figure 2C). By contrast, salt elution fractions (IPS30S1, IPS60S1, IPS60S2, and IPS80S1) had lower polysaccharides content and yields (Figure 2C). A similar trend was observed for the extraction yield, as the water-eluted (IPS30W, IPS60W, and IPS80W) had higher yields than the salt-eluted (*p* < 0.05). Water fractionation of polysaccharide from *P. igniarius* mycelia was more effective than salt fractionation (Figure 2C) because most polysaccharides have a high affinity for water due to their numerous hydrophilic hydroxyl groups, making them readily soluble in pure water [17,39,40]. However, adding salt can disrupt these water–polysaccharide interactions, leading to precipitation at lower concentrations compared with other molecules with less hydrophilic sites, and easily forms co-precipitation [41]; essentially, the salt competes with the polysaccharide for water molecules, causing it to precipitate out.

Considering this, IPS30W, IPS60W, and IPS80W were selected to continue purification. After repeated purification by Sephacryl^TM^ S-400 column (Figure 1, purification flow section), the purified polysaccharides were obtained: IPSW-2 and IPSW-3 from IPS60W, with yields of 8.0% and 11.01%, respectively; and IPSW-4 from IPS80W, with a yield of 15.83% (Figure 2D), which was calculated according to IPS30W, IPS60W, and IPS80W, respectively. In addition, we measured the protein content in each fractionation stage (Figure 2B,C), and it was observed that the protein content reduced from the first fractionation stage (~2% as shown in Figure 2B) to ~0.15% during purification using a DEAE-52 cellulose column (Figure 2C), and no protein was present in the final products, IPSW-2 to 4, when Sephacryl^TM^ S-400 gel-filtration column chromatography was applied. A slightly higher protein content in the crude polysaccharides (Figure 2B) and in the polysaccharides from DEAE-52 cellulose column chromatography (Figure 2C) may be because of the hydrolysis of the amide bonds of proteins, and the results were in tandem with Fructus mori [42] and blackberry fruit polysaccharides [37].

According to our previous report, in which the physicochemical properties of IPSW-1 to IPSW-4 were analyzed [14], the results of size-exclusion chromatography coupled with multi-angle laser light scattering and refractive index detection indicated that IPSW-2, IPSW-3, and IPSW-4 are homogenous polysaccharides, with the Mw of 17.7 kDa, 15.1 kDa, and 21.7 kDa, respectively. The findings of the component analysis (carbohydrate, protein, nucleic acid, and uronic acid) revealed that IPSW-2, IPSW-3, and IPSW-4 are 94.08% and 5.84%, 92.15% and 7.82%, and 89.33% and 10.98%, respectively, with no protein and nucleic acid. Furthermore, the results of the monosaccharide composition analysis in our previous study [14] indicated that IPSW-2 and IPSW-3 are composed of only glucose, a pyran-type homopolysaccharide with α-configuration, whereas the rhamnose, xylose, mannose, glucose, and galactose (in a molar ratio of 1.29:1.21:1:43.86:1.86) were detected in IPSW-4, which showed that IPSW-4 is a heteropolysaccharide with α-configuration.

### 3.2. Structure Characterization of Homogeneous Polysaccharides

#### 3.2.1. Periodate Oxidation and Smith Degradation

Periodate oxidation and Smith degradation were performed to identify linkages among the four polysaccharides [22]. The periodate oxidation of IPSW-2 to IPSW-4 was carried out in the dark; the reaction completion was detected using absorbance at 223 nm and reached stable absorbance after 168 h. The consumed periodate and the yielded formic acid per mole of sugar residue calculated were 0.346 mol and 0.0075 mol for IPSW-2, 0.306 mol and 0.114 mol for IPSW-3, and 0.408 mol and 0.048 mol for IPSW-4 (Table 1). The value of production of formic acid was between 0 and ~1 mol/mole of sugar residue, indicating the existence of (1→)-linked or (1→6)-linked linkages of monosaccharide periodate [27,43], as shown in Table S1.

Meanwhile, formic acid production was less than half the consumption of periodate, signifying that some linkages in four polysaccharides, such as (1→2,6)-linked, (1→2)-linked, (1→4,6) or (1→4)-linked sugar residue only consumed the periodic acid without producing formic acid [44]. The periodate consumption that was < 1 mol/mole of sugar residue showed the possible existence of (1→3), (1→3,6), (1→2,3), (→2,4), (1→3,4), or (1→2,3,4)-association, which neither consumed periodic acid nor created formic acid throughout the oxidation process [45].

After further Smith degradation of the periodate-oxidized IPSW-2 to IPSW-4, the hydrolysates were analyzed by GC-FID after aldononitrile acetate derivatization (Figure S1 and Table 1). Because of the variable condensation spots between sugar bonds, numerous products were formed following oxidation, which were reduced to stable polyhydroxy compounds [22].

The glycerol released after Smith degradation indicated the presence of (1→6), (1→2), or (1→2,6) links in the IPSW-2, IPSW-3, and IPSW-4, respectively [43,44]. Also, the GC detected no erythritol, reflecting no (1→4) and (1→4,6)-linked sugar residue [43,44].

#### 3.2.2. Methylation Analysis Using FTIR and GC-MS Analysis

**Figure 3:**
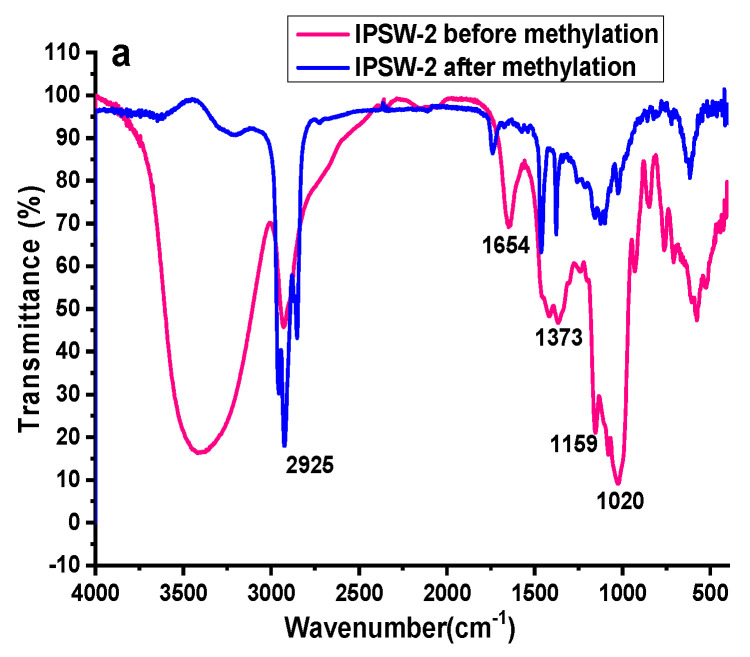

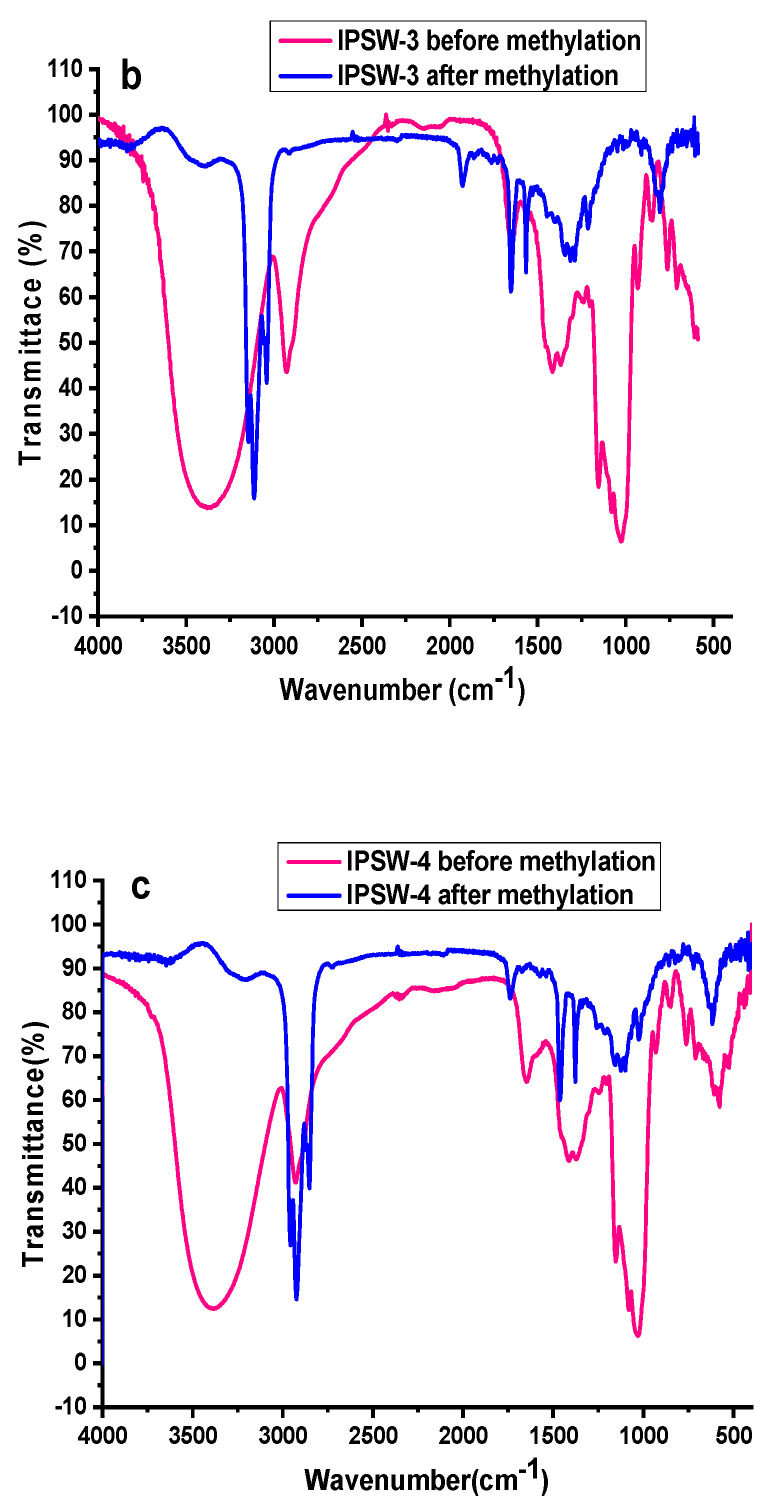
FT-IR spectrum of IPSW-2 (**a**), IPSW-3 (**b**), and IPSW-4 (**c**) before methylation and after methylation.

To further establish the polysaccharide’s monomer units and linkage chain, IPSW-2 to IPSW-4 were methylated with methyl iodide. The FT-IR spectrum monitored the methylation extent [46]. After three rounds of methylation reactions, fully methylated IPSW-2 to IPSW-4 were obtained (Figure 3). As shown in Figure 3, after three rounds of methylation, the absorption peak at around 3400 cm^−1^ of IPSW-2 to IPSW-4 disappeared, while the methyl absorption peak at 2900 cm^−1^ significantly increased, indicating that IPSW had been completely methylated and could be analyzed in the next step.

Then, these fully methylated polysaccharides were hydrolyzed by acid and then reduced and converted into alditol acetates [47]; a series of methylated derivatives were found according to the GC-MS results (Table 2, Figures S2–S4), and the glycosidic linkage patterns and monosaccharide residues’ molar ratios were evaluated. The derivatives of three polysaccharides were identified as 2,3,4,6-Me_4_-Glc*p*; 2,3,4-Me_3_-Glc*p*; 2,6-Me_2_-Glc*p*; 2,4-Me_2_-Glc*p*; and 3,4-Me_2_-Rha*p*. There were the forms of terminal; 1,6-linked; 1,3,4-linked; and 1,3,6-linked Glc*p* residues and the form of 1,2-linked Rha*p* residues.

According to the methylation results and monosaccharide composition, it was suggested that IPSW-2 and IPSW-3 were all glucan, with the main chain consisting of (1→6)-linked, (1→3,4)-linked, and (1→3, 6)-linked. IPSW-4 was a heteropolysaccharide mainly comprising glucose (~90%). So, it was inferred that the main chain comprises (1→6)-linked-glucose and (1→3,4)-linked-glucose, and the branch chain may consist of (1→2)-linked-Rha*p*.

### 3.3. NMR Analysis

**Figure 4:**
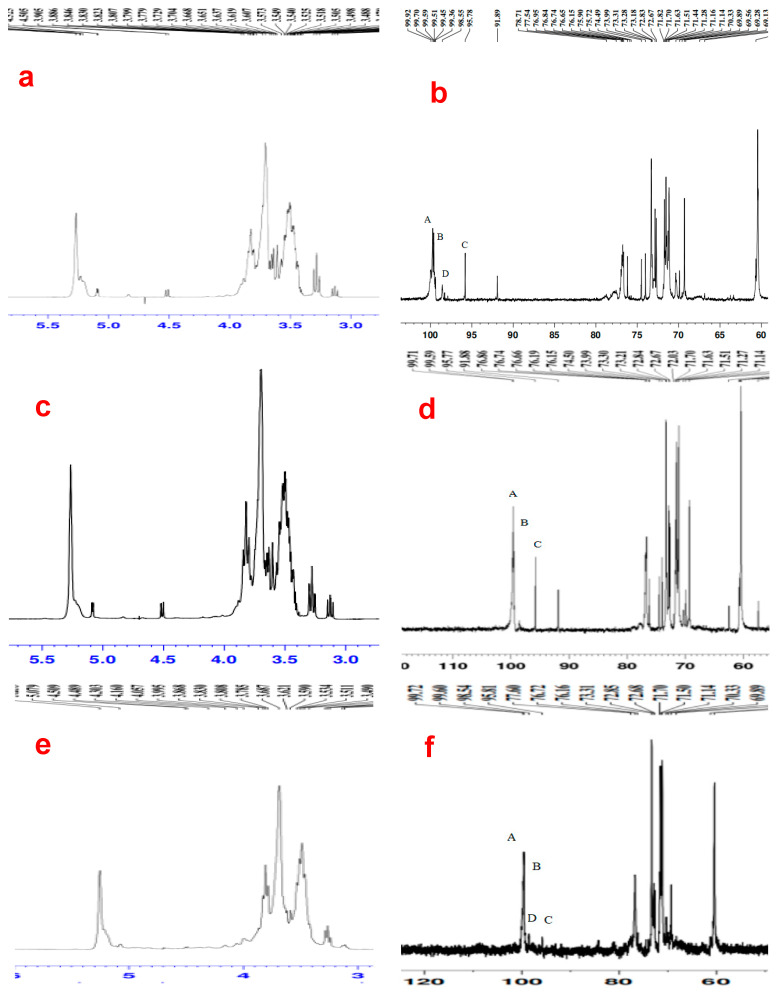
a–b: NMR spectra of IPSW-2 using (**a**) ^1^H NMR and (**b**) ^13^C NMR. c–d: NMR spectra of IPSW-3 using (**c**) ^1^H NMR and (**d**) ^13^C NMR. e–f: NMR spectra of IPSW-4 using (**e**) ^1^H NMR and (**f**) ^13^C NMR. Note: For (**b**): A, B, C, and D are 99.70, 99.62, 95.84, and 98.55 ppm, respectively; for (**d**): A, B, and C are 99.71, 99.59, and 95.77 ppm, respectively; and for (**f**): A, B, C, and D are 99.72, 99.60, 95.81, and 98.54 ppm, respectively.

Since chemical methods such as Smith degradation, periodic acid oxidation, and methylation analysis cannot completely determine the connection mode between individual sugar residues, nuclear magnetic resonance (NMR) was used to determine further the connection mode and structure of the monosaccharide residues in polysaccharides, as NMR is a critical spectroscopic tool to identify the molecular structures of polysaccharides and glycosidic linkages (i.e., α and/or β) [48]. ^1^H NMR mainly solves the problem of glycosidic bond configuration in polysaccharides. Generally, the δ value of anomeric hydrogen > 5 ppm is an α type, and the δ value < 5 ppm is a β type [43]. The ^13^C-NMR spectrum can assess the proportion of sugar residues, the number of anomeric carbons, and the sugar ring conformation, and most of the signals of anomeric carbons appear between 90 and 110 ppm [49]. ^13^C NMR chemical shifts are generally wider, between 0 and 200 ppm, and the C1 chemical shift of the α type linkage was 1.5–3.0 lower than that of the β type. The chemical shift of the α type was 97–101 ppm and that of the β type was 103–105 ppm. Regarding the NMR analysis of IPWS-2 (Figure 4a,b), it can be seen that the ^1^H NMR pattern of IPSW-2 (Figure 4a) showed no peak near δ5.4 ppm, indicating that IPSW-2 is a pyranose sugar. Four resonance peaks of anomeric hydrogen appeared between δ5.0 ppm and 5.4 ppm, namely, at 5.266, 5.229, 5.094, and 5.085 ppm, indicating that the glycosidic bond configuration of IPSW-2 is α type. The overlapping resonance peaks between δ3.0 and 4.0 ppm (Figure 4a) do not provide accurate information for analysis, but the chemical shift of hydrogen protons (H-1) on the anomeric carbon (C-1) is in the lower field, mostly at 4.5–5.5 ppm, which eases structural analysis [33,50]. Li et al. [51] had a comparable scenario, making it challenging to identify the anomeric proton signals merged with the HOD signal. Figure 4b shows the ^13^C NMR spectrum of IPSW-2. According to Zhou et al. [33] and Huang et al. [50], if the signal shift lies between 103 and 110 ppm, it can be ascribed to a β type glycosidic bond, and between 90 and 103 ppm to an α type glycosidic bond. Four anomeric carbon signals of IPSW-2 appeared between δ95 and 100 ppm (Figure 4b), which are 99.70 (labeled A), 99.59 (labeled B), 95.78 (labeled C), and 98.55 (labeled D), respectively, and the chemical shifts of the anomeric carbons were from 95 to 110 ppm [28,52]. The signal values of these four anomeric carbons were all < 100 ppm, and there was no signal between δ82 and 84 ppm, further indicating that there is no furanose in IPSW-2 and that the glycosidic bond configuration is α type [53]. Comparing our results with the NMR spectroscopic assignments for α-D-glucans by Yao et al. [54], Zhang et al. [52], Aburas et al. [55], Shi et al. [28], Ishurd et al. [56], and Mutaillifu et al. [29], the signals at δ99.70 (anomeric carbon A) and δ69.56 ppm were ascribed to C-1 and C-6 of α-1,6-Glc*p*; the signals at δ99.59 (anomeric carbon B) and δ60.65 ppm may be contributed by C-1 and C-6 of α-T-Glc*p*; the chemical shift values at δ98.55 (D), δ76.95, and δ69.89 ppm were ascribed to C-1, C-3, and C-6 of α-1,3,6-Glcp; and the signals at δ95.78 (C), δ77.54, and δ76.74 ppm were ascribed to C-1, C-3, and C-4 of α-1,3,4-Glc*p*, respectively (Figure 4b).

For IPSW-3, the ^1^H NMR spectrum (Figure 4c) showed no peak near δ5.4 ppm, indicating that IPSW-3 is a pyranose sugar. Four resonance peaks of anomeric hydrogen appeared between δ5.0 ppm and 5.4 ppm, namely 5.264, 5.233, 5.088, and 5.079 ppm, indicating that the glycosidic bond configuration of IPSW-3 is α type [49]. The overlapping resonance peaks between δ3.0 and 4.0 ppm did not provide accurate information. For the ^13^C NMR spectrum of IPSW-3 (Figure 4d), three anomeric carbon signals appeared between δ95 and 100 ppm, which are 99.71 (A), 99.59 (B), and 95.77 (C), respectively, further indicating that IPSW-3 does not contain furanose and that the glycosidic bond configuration is α type. Based on references Yao et al. [54], Zhang et al. [52], Aburas et al. [55], Shi et al. [28], Ishurd et al. [56], and Mutaillifu et al. [29], the anomeric carbon signals at δ99.71 (A), 99.59(B), and 95.77 (C) ppm were ascribed to anomeric carbon (C-1) of α-1,6-Glc*p*, α-T-Glc*p,* and α-1,3,4-Glc*p*. The signals at δ69.29 and δ60.65 ppm were ascribed to C-6 of α-1,6-Glc*p* and α-T-Glc*p*, respectively, and the signal at δ76.74 ppm was ascribed to C-4 of α-1,3,4-Glc*p*. The signal values at δ76.86 and δ69.90 ppm were ascribed to C-3 and C-6 of α-1,3,6-Glc*p*, and the anomeric carbon signal of α-1,3,6-Glc*p* cannot be found in Figure 4d.

Regarding IPWS-4, the ^1^H NMR and ^13^C NMR spectra (Figure 4e,f) are similar to those of IPWS-2 and IPWS-3, and all the data indicated that the glycosidic bond configuration of IPSW-4 was α type. In the ^13^C NMR spectrum of IPSW-4 (Figure 4f), there were four signals of anomeric carbons between δ95 and 100 ppm, which are δ99.72(A), δ99.60(B), δ98.54(C), and δ95.81(D), respectively. According to the references of Yao et al. [54], Zhang et al. [52], Aburas et al. [55], Huang et al. [50], Chen et al. [57], Shi et al. [28], and Mutaillifu et al. [29], the signals at δ99.72(A) and δ69.29 ppm were ascribed to anomeric carbons (C-1) and C-6 of α-1,6-Glc*p*. The signals at δ99.60(B) and δ60.39 ppm were ascribed to anomeric carbons (C-1) and C-6 of α-T-Glc*p*, respectively. The anomeric carbon δ95.81(D) ppm was ascribed to C-1 of α-1,3,4-Glc*p*, and its C-3 and C-4 signals may be present at δ77.60 ppm and δ76.72 ppm. And the signal at δ98.54(C) was ascribed to anomeric carbons (C-1) of α-1,2-Rha*p*.

Comparing the NMR data of IPWS-2 to 4, the signals at (A), (B), and (C) for the three products IPSW-2 to 4 (Figure 4b,d,f) were ascribed to anomeric (C-1) carbons of the (1→6)-linked α-glucose, (1→)-linked α-glucose (B), and (1→3,4)-linked α-glucose (C), respectively.

Based on the infrared spectrum, periodic acid oxidation, Smith degradation, methylation, and NMR analysis of IPSW-2 to 4, their structure can be preliminarily speculated.

The molecular structure of IPSW-2 was determined: the main chain is Glc*p*(1→6), Glc*p*(1→3,4), and Glc*p*(1→3,6) and the side chain is Glc*p*(1→); the non-reducing end is Glc*p*; and each glycoside mainly exists in the form of α-pyranose. The molar ratio of Glc*p*(1→6), Glc*p*(1→), Glc*p*(1→3,4), and Glc*p*(1→3,6) was about 3.39:3.09:1.78:1.0 (Table 2). It is speculated that the repeating units of IPSW-2’s primary structure might be as follows: 

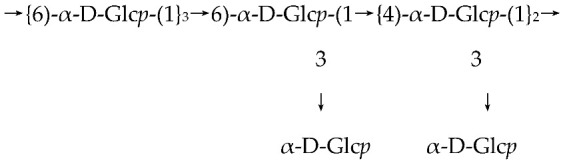



For IPSW-3, the main chain is Glc*p*(1→6), Glc*p*(1→3,4), and Glc*p*(1→3,6); the non-reducing end is Glc*p*; and each glycoside mainly exists in the form of α-pyranose. The molar ratio of Glc*p*(1→6), Glc*p*(1→), Glc*p*(1→3,4), and Glc*p*(1→3,6) of IPSW-3 was about 7.94:3.56:3.63:1.0 (Table 2). The repeating units of IPSW-3’s primary structure are speculated as follows:

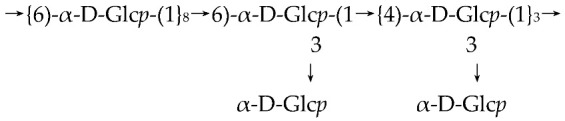



Finally, for IPSW-4, the main chain is Glc*p*(1→6) and Glc*p*(1→3,4), the branched chain might be Rha*p* (1→2), the non-reducing end is Glc*p*, and each glycoside mainly exists in the form of α-pyranose. The repeating units of IPSW-4’s primary structure are speculated as follows, per the molar ratio of each residue in Table 2.

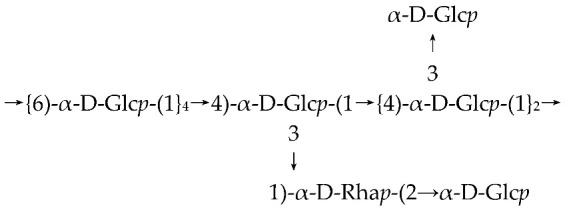



The 2D NMR tests assist to assign all nuclei in addition to the connection between the sugar moieties, therefore predicting the sequence and, thus, the polysaccharides’ molecular structures [48,58]. Therefore, future studies should use 2D NMR, as the significant advantage of 2D NMR over 1D NMR is the capability to differentiate between the overlapping signals in larger molecules.

### 3.4. Conformational Characteristics of Polysaccharides in Sodium Hydroxide Solution

**Figure 5:**
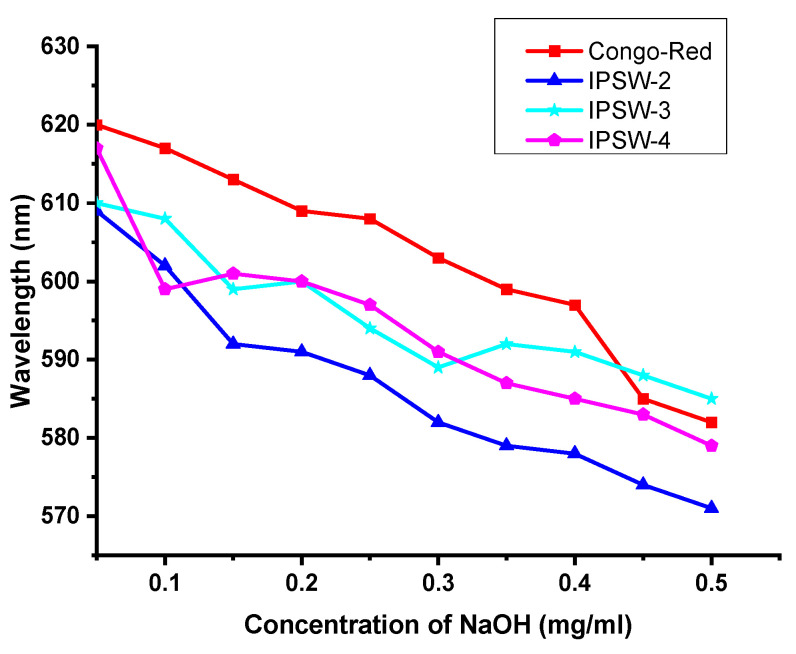
Dependence of the λmax of Congo red analysis of various polysaccharide complexes at various concentrations of sodium hydroxide.

Polysaccharides with a triple helical structure can form complexes with Congo red in weakly alkaline solutions. These complexes cause a red shift in the maximum absorption wavelength compared with pure Congo red solution. However, in strong alkaline solutions, the triple helical structure is disrupted, leading to a weakening of the red-shift effect in the Congo red–polysaccharide complex [59,60]. Whether the polysaccharides have a triple helix conformation was assessed by observing the red-shift phenomenon in the experiment and the degree of decrease in the maximum absorption wavelength under alkaline conditions [61]. At 0–0.5 mol/L NaOH, it can be determined whether the researched polysaccharide has a triple-helix structure based on the change in λmax of the complex produced by the studied polysaccharide and Congo red [60]. The Congo red analysis results (Figure 5) showed that the maximum absorbance of IPSW-2 to IPSW-4 and Congo red mixture was not greater than that of Congo red alone in the range of NaOH concentration (0 to 0.5 mg/mL), which indicates that no polysaccharide–Congo red complex was formed at the time. Furthermore, there was no triple helical structure in IPSW-2 to 4 [62].

Under alkaline conditions, Congo red can form complexes with polysaccharides containing helical conformations, and the maximum absorption wavelength of the complex will also change with different concentrations of sodium hydroxide. If the polysaccharide contains a regular triple-helix structure, generally within the range of low sodium hydroxide concentrations, the complex’s maximum absorption wavelength will shift to longer wavelengths. As the concentration of the alkali solution gradually increases, the maximum absorption wavelength of the solution will decrease due to the unwinding of the triple helix and the formation of complexes. The change in the maximum wavelength of the IPSW-2 Congo red solution with sodium hydroxide concentration is shown in Figure 5. It can be seen from the figure that the homogeneous polysaccharide IPSW-2 cannot red-shift the maximum absorption wavelength of Congo red at low concentrations, so it does not have a regular triple-helix structure. A similar trend was observed for IPSW-3 and IPSW-4 (Figure 5). In general, non β-(1→3)-glucans or other polysaccharide chains cannot give this color reaction with Congo red. Nonetheless, the length of the main and branch chain, and the presence or absence of the branch chain of β-(1→3)-glucans, can impact the stability of triple-helix strands [60]. In future studies, a circular dichroism test may be supplemented to confirm the absence of trihelix structures

### 3.5. Antioxidant Activity of IPS30, IPS60, and IPS80

**Figure 6:**
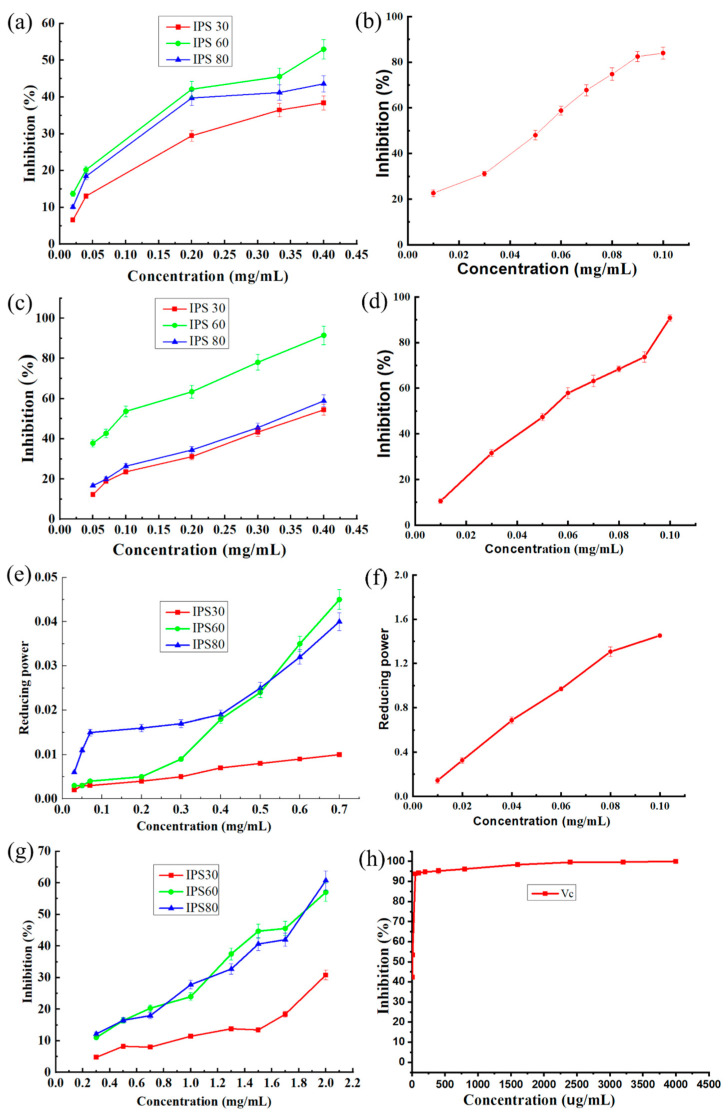
a–b: Scavenging activity (% inhibition) of (**a**) IPS30, IPS60, and IPS80 and (**b**) vitamin C on superoxide anion radicals. c–d: Scavenging activity of (**c**) IPS30, IPS60, and IPS80 and (**d**) vitamin C on hydroxyl radicals. e–f: The reduction power of (**e**) IPS30, IPS60, and IPS80 and (**f**) vitamin C radical. g–h: Scavenging activity of (**g**) IPS30, IPS60, and IPS80 and (**h**) Vitamin C on DPPH.

Free radicals can react with nearly every biomacromolecule in living cells, causing significant damage to the nearby biomolecules [63]. As a result, effective scavenging of free radicals is critical for antioxidant defense in cells and food systems. The ability of IPS30, IPS60, and IPS80 to scavenge superoxide anions is shown in Figure 6a. It can be seen that the ability of IPS30, IPS60, and IPS80 to scavenge superoxide anions showed a trend of first increasing rapidly and then increasing slowly as the concentration increased. When the crude polysaccharide concentration was 0.40 mg/mL, the scavenging rates of superoxide anions by IPS30, IPS60, and IPS80 reached 38.39%, 52.98%, and 43.58%, respectively. The IC_50_ of vitamin C (Vc) was 0.0529 mg/mL. The IC_50_ of IPS60 was 0.37 mg/mL, similar to burdock crude polysaccharides’ ability to scavenge superoxide anions (IC_50_ was 0.34 mg/mL) [64]. The polysaccharide structure of uronic acid contributed significantly to the radical scavenging [23]. The three crude polysaccharides of the *P. igniarius* mycelium were all more capable of scavenging superoxide anions than the polysaccharides of Phyllostachys (13.8%), floribunda (15.1%), and mushroom (14.8%) at the same concentration (0.40 mg/mL) [64]. It can be seen that the three types of crude polysaccharides of *P. igniarius* mycelium all had good scavenging abilities for superoxide anions, and the scavenging ability of IPS60 was the strongest, followed by IPS80 and then IPS30, which was the weakest. Due to their extremely strong oxidizing properties, hydroxyl free radicals can react with all cells, causing great damage to the body. This study used the H_2_O_2_-Fe^2+^ reaction system as the generated hydroxyl radicals to oxidize Fe^2+^ into Fe^3+^. The color of the solution also changed with the reaction, showing different absorption intensities at 536 nm. Active substances that can scavenge hydroxyl radicals were added. Finally, its absorption intensity at 536 nm could be changed, and the ability to scavenge hydroxyl radicals could be judged. The ability of IPS30, IPS60, and IPS80 to scavenge hydroxyl radicals is shown in Figure 6c. The ability of the three crude polysaccharides to scavenge hydroxyl radicals increased with the increase in polysaccharide concentration. At 0.40 mg/mL, the scavenging rate of IPS60 for hydroxyl radicals was as high as 91.46%, while the scavenging rates of IPS30 and IPS80 were 54.44% and 58.89%, respectively. The IC_50_ of Vc was 0.0527 mg/mL and those of IPS30, IPS60, and IPS80 were 0.36 mg/mL, 0.09 mg/mL, and 0.33 mg/mL, respectively. Among them, the IC_50_ of IPS60 (0.09 mg/mL) was close to that of Vc, showing a good ability to scavenge hydroxyl radicals. Xing et al. [65] found that lower-MW chitosan fractions (9 kDa) had much stronger hydroxyl radical scavenging efficacy than high-molecular-weight fractions (760 kDa). Furthermore, Xie et al. [66] found that the hydroxyl group in the polysaccharide unit can scavenge the free radical by reacting with it using the standard hydrogen-abstraction reaction. In research by Zhang et al. [23], PIPS with larger levels of low-molecular-weight fractions and hydroxyl groups demonstrated greater hydroxyl radical scavenging activity, which is consistent with our results. The hydroxyl radical scavenging capabilities of IPS30 and IPS80 were similar to the IC_50_ value of burdock crude polysaccharide (0.28 mg/mL) for scavenging hydroxyl radicals and the IC_50_ value (0.358 mg/mL) of the *Cordyceps militaris* fruiting body polysaccharide [67]. Compared with the results of scavenging superoxide anions, the three types of *P. igniarius* mycelium crude polysaccharides had stronger hydroxyl radical scavenging effects than their superoxide anion scavenging effects. IPS60 had the strongest scavenging ability among the three crude polysaccharides, while IPS30 had the weakest.

The reducing power measurement results of IPS30, IPS60, and PS80 are shown in Figure 6e. IPS60 and IPS80 showed a sharp enhancement trend with increased polysaccharide concentration. In contrast, the enhancement speed of IPS30 was relatively slow, indicating that IPS60 and IPS80 had strong reducing ability. When the polysaccharide concentration was 0.40 mg/mL, the reducing powers of IPS30, IPS60, and IPS80 were 0.007, 0.018, and 0.019, respectively. When the concentration of Vc was 0.10 mg/mL (Figure 6f), its reducing power reached 1.452, indicating that the crude polysaccharide of *P. igniarius* mycelium had a weak scavenging ability. This may be because polysaccharides are macromolecular substances with relatively few reducing ends.

The ability of IPS30, IPS60, and IPS80 to remove DPPH· is shown in Figure 6g. The DPPH-scavenging ability of IPS30, IPS60, and IPS80 showed a gradually increasing trend with the increase in polysaccharide concentration. When the concentration was 0.40 mg/mL, the scavenging capabilities of IPS30, IPS60, and IPS80 for DPPH· were 6.72%, 13.62%, and 14.38%, respectively; the IC_50_ of IPS60 and IPS80 were 1.82 mg/mL and 1.83 mg/mL, respectively, while for Vc, the IC_50_ was 2.35 mg/mL (Figure 6h), indicating that the crude polysaccharide of *P. igniarius* mycelium had a weak scavenging ability of DPPH·. It may be because Vc itself has strong antioxidant properties, and DPPH· is soluble in ethanol, but polysaccharides generally have poor solubility in organic solvents, so polysaccharide molecules cannot fully contact DPPH. The IC_50_ of the high-grade edible fungi boletus crude polysaccharide, morel crude polysaccharide, and matsutake crude polysaccharide for DPPH· scavenging are 1.48 mg/mL, 1.76 mg/mL, and 2.14 mg/mL, respectively [67], which is similar to the scavenging effect of the *P. igniarius* mycelium crude polysaccharides IPS60 and IPS80 on DPPH· in this experiment.

Comparing the four antioxidant experimental results of *P. igniarius* mycelium polysaccharides, the three crude polysaccharides of *P. igniarius* mycelium have the strongest ability to scavenge hydroxyl radicals, followed by their ability to scavenge superoxide anions. The reducing power and DPPH scavenging power of polysaccharides were relatively weak. Future studies should investigate in vivo antioxidant studies. Moreover, the mechanisms by which the identified polysaccharides exert their antioxidant effects should be investigated in future studies as this would provide more insight into how these compounds could be harnessed in practical applications.

## 4. Conclusions

In conclusion, the water elution fractions of the IPS30W, IPS60W, and IPS80W polysaccharides content was 79.05%, 68.25%, and 62.06%, with yields of 25.07%, 21.38%, and 20.34%, respectively. By contrast, the salt elution fractions (IPS30S1, IPS60S1, IPS60S2, and IPS80S1) had lower polysaccharides content and yield. Hence, water elution fractions were selected for purification. After repeated purification, the four purified polysaccharides (IPSW-2 to 4) were obtained with yields of 8% to 15.83%. The IPSW-2 to 4 structures were elucidated, and they showed no triple helical conformation. Based on periodate oxidation, Smith degradation, methylation analysis, and ^1^H and ^13^C NMR spectroscopy data, the primary structures of IPSW-2 to IPSW-4 are an α-D-glucan with a repeating backbone composed of glucopyranose. The various antioxidant activities showed that IPS30W, IPS60W, and IPS80W were all more capable of scavenging superoxide anions than the polysaccharides of Phyllostachys (13.8%) and floribunda (15.1%) at the same concentration (0.40 mg/mL). This will serve as a guide for the development of functional foods. However, this research is specific to the *P. igniarius* mycelium strain No. 5.95 cultivated via liquid fermentation. This may limit the generality of the results to other strains or cultivation methods, which may produce polysaccharides with different structures and activities. Hence, future studies could compare various strains and their effect on fractionation, structure, and bioactivities. Future studies should use 2D NMR, as the significant advantage of 2D NMR over 1D NMR is the ability to distinguish between the overlapping signals in larger molecules.

## Figures and Tables

**Table 1:** The results of periodate oxidation/Smith degradation of IPSW-2, IPSW-3, and IPSW-4.

Standard/Samples	Consumption of Periodate (mol/mol Glc)	Yield of Formic Acid (mol/mol Glc)	Retention Time in GC of Products of Smith Degradation (min)	Products of Smith Degradation
Glycerol	n	n	27.135	/
Erythritol	n	n	18.233	/
IPSW-2	0.346	0.0075	27.170	Glycerol
IPSW-3	0.306	0.114	27.183	Glycerol
IPSW-4	0.408	0.048	27.179	Glycerol

**Table 2:** The methylation analysis results of IPSW-2, IPSW-3, and IPSW-4.

	Methylated Sugar	Molar Ratio (mol%)	Linkage Type	Major Mass Fragments (*m*/*z*)
IPSW-2	2,3,4,6-Me_4_-Glc*p*	3.09	Terminal	43,71,87,101,117,129,145,161,205
	2,4-Me_2_-Glc*p*	1.00	1,3,6-linked-Glc*p*	43,71,89,101,117,162,261
	2,3,4-Me_3_-Glc*p*	3.39	1,6-linked-Glc*p*	43,71,87,101,117,129,161,189,233
	2,6-Me_2_-Glc*p*	1.78	1,3,4-linked-Glc*p*	43,71,87,101,117,129,143,231,305
IPSW-3	2,3,4,6-Me_4_-Glc*p*	3.56	Terminal	43,71,87,101,117,129,145,161,205
	2,4-Me_2_-Glc*p*	1.00	1,3,6-linked-Glc*p*	43,71,89,101,117,162,261
	2,3,4-Me_3_-Glc*p*	7.94	1,6-linked-Glc*p*	43,71,87,101,117,129,161,189,233
	2,6-Me_2_-Glc*p*	3.63	1,3,4-linked-Glc*p*	43,71,87,101,117,129,143,231,305
IPSW-4	2,3,4,6-Me_4_-Glc*p*	2.59	Terminal	43,71,87,101,117,129,145,161,205
	2,3,4-Me_3_-Glc*p*	3.87	1,6-linked-Glc*p*	43,71,87,101,117,129,161,189,233
	2,6-Me_2_-Glc*p*	3.18	1,3,4-linked-Glc*p*	43,71,87,101,117,129,143,231,305
	3,4-Me_2_-Rha*p*	1	1,2-linked-Rhap	57,71,87,131,284,328

## Data Availability

The original contributions presented in the study are included in the article/Supplementary Materials, further inquiries can be directed to the corresponding author.

## Supplementary Materials

The following supporting information can be downloaded at: https://www.mdpi.com/article/10.3390/foods13223581/s1. Figure S1: GC chromatogram of IPSW Smith degradation product. Figure S2: Total ion flow chromatogram of alditol acetate derivatives after fully methylated IPSW-2 hydrolysis reduction. Figure S3: Total ion flow chromatogram of alditol acetate derivatives after fully methylated IPSW-3 hydrolysis reduction. Figure S4: Total ion flow chromatogram of alditol acetate derivatives after fully methylated IPSW-4 hydrolysis reduction. Table S1. The results of periodate oxidation/Smith degration of different glycosyl linkage.
