# Supplementary material for: Water-Soluble Intracellular Polysaccharides (IPSW-2 to 4) from Phellinus igniarius Mycelia: Fractionation, Structural Elucidation, and Antioxidant Activity
Source: Foods. 2024 Nov 9;13(22):3581. doi: 10.3390/foods13223581 (PMC11593105; doi:10.3390/foods13223581)
Supplement: Supplementary file 1 [file foods-13-03581-s001.zip › foods-3288331-supplementary.pdf]

## Supplementary data

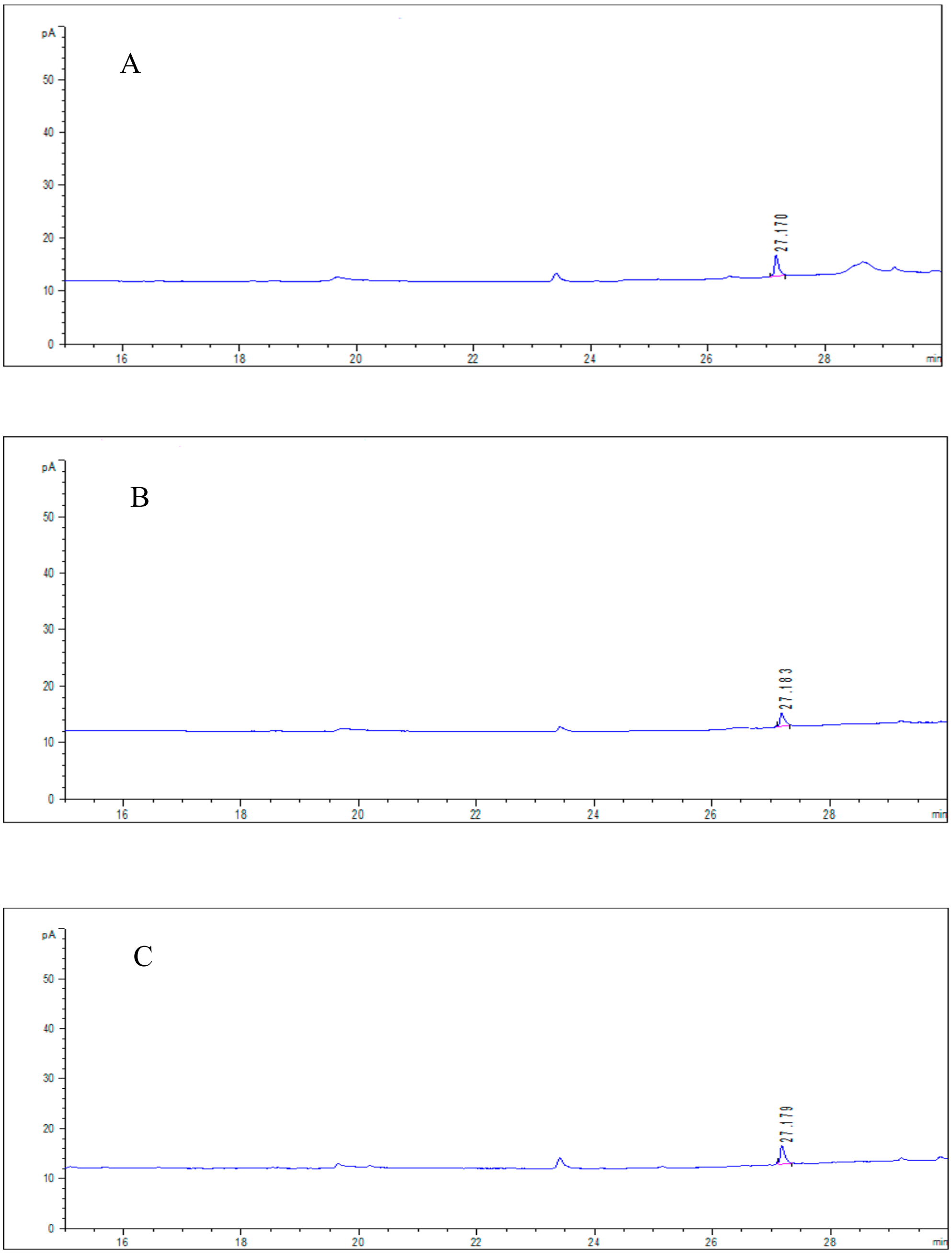


**Fig. S1.** GC chromatogram of Smith degradation products of IPSW-2 (A), IPSW-3(B) and IPSW-4(C).

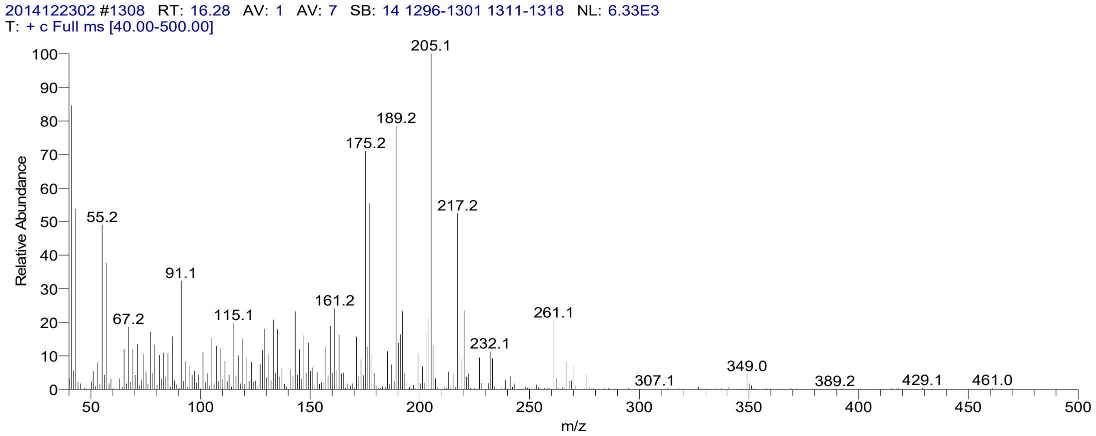
2014122302 #1308 RT: 16.28 AV: 1 AV: 7 SB: 14 1296-1301 1311-1318 NL: 6.33E3
T: + c Full ms [40.00-500.00]
Relative Abundance
m/z
205.1
189.2
175.2
217.2
55.2
91.1
67.2
115.1
161.2
261.1
232.1
307.1
349.0
389.2
429.1
461.0

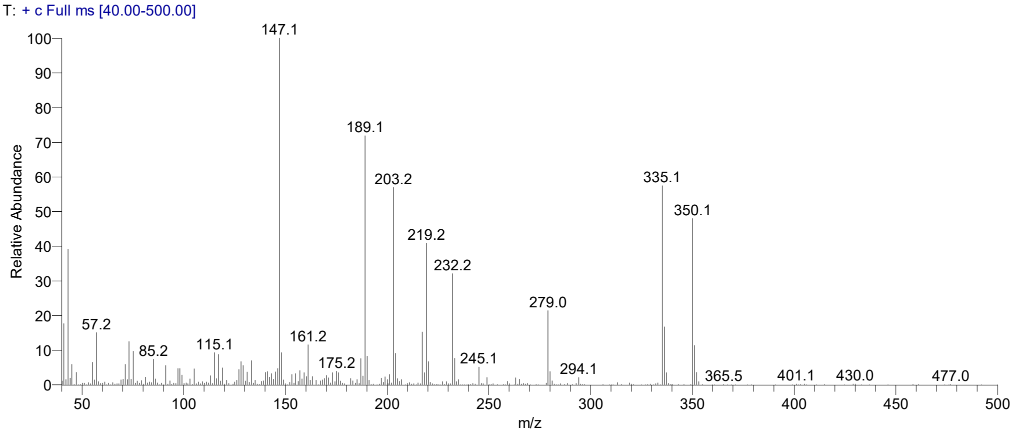
T: + c Full ms [40.00-500.00]
Relative Abundance
m/z
147.1
189.1
203.2
335.1
350.1
219.2
232.2
279.0
57.2
85.2
115.1
161.2
175.2
245.1
294.1
365.5
401.1
430.0
477.0

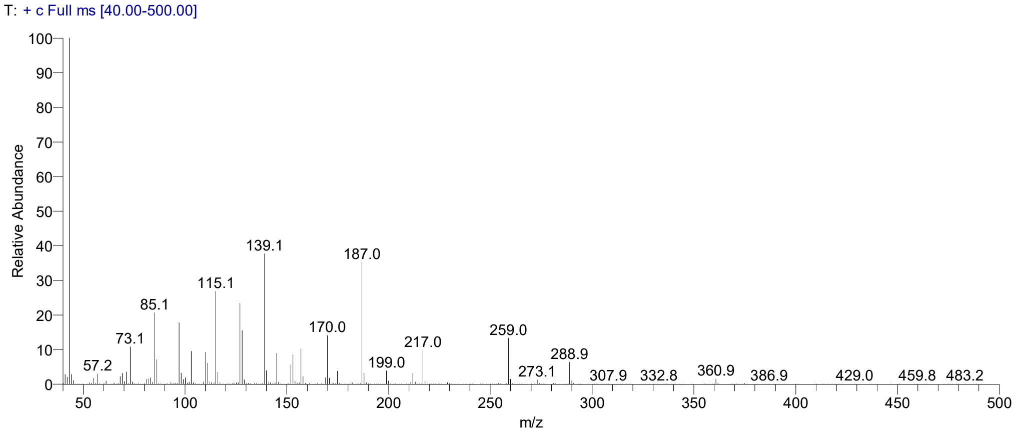
T: + c Full ms [40.00-500.00]
Relative Abundance
m/z
139.1
187.0
115.1
85.1
73.1
57.2
170.0
199.0
217.0
259.0
273.1
288.9
307.9
332.8
360.9
386.9
429.0
459.8
483.2

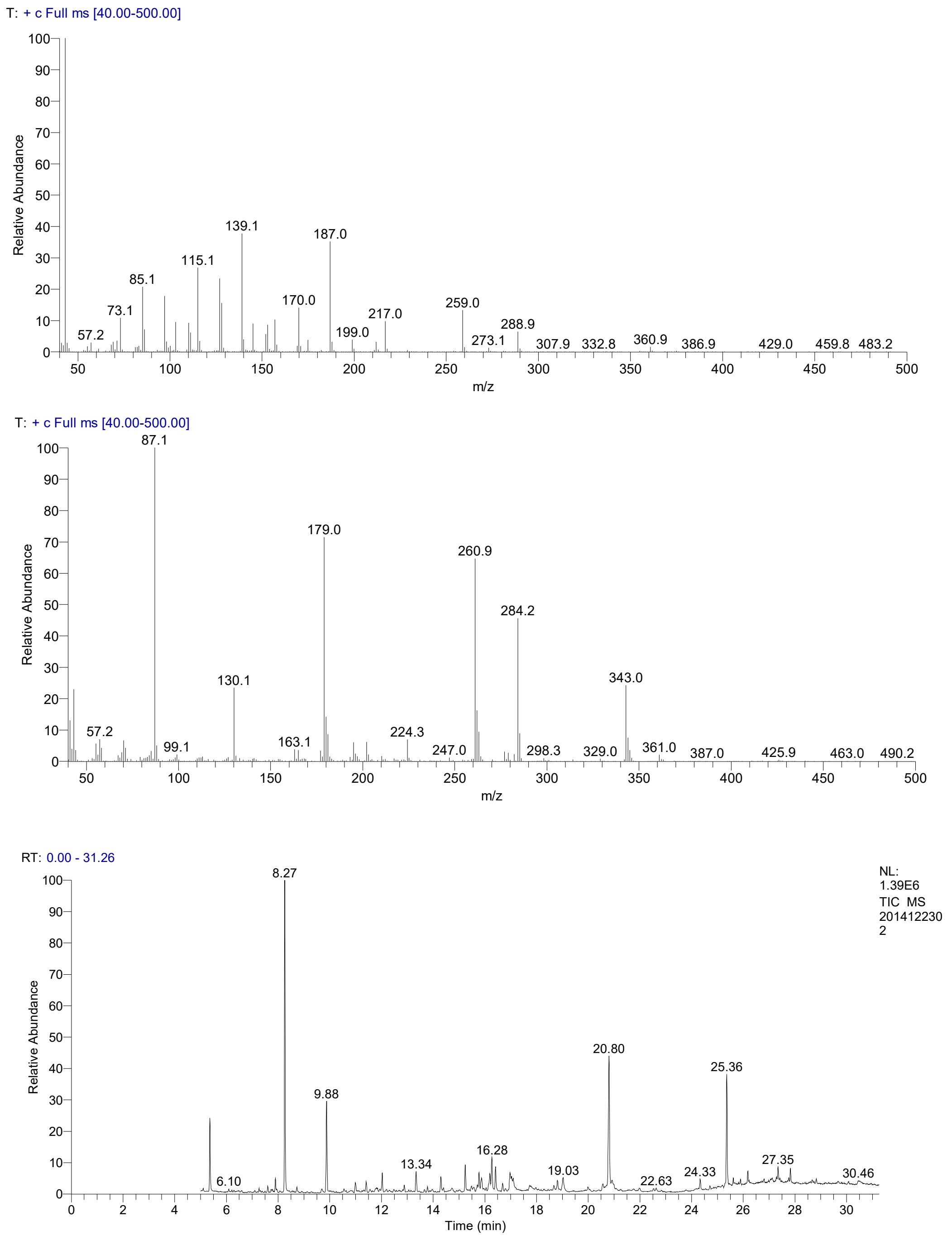


**Fig S2.** Total ion flow chromatogram of alditol acetate derivatives after fully methylated IPSW-2 hydrolysis reduction

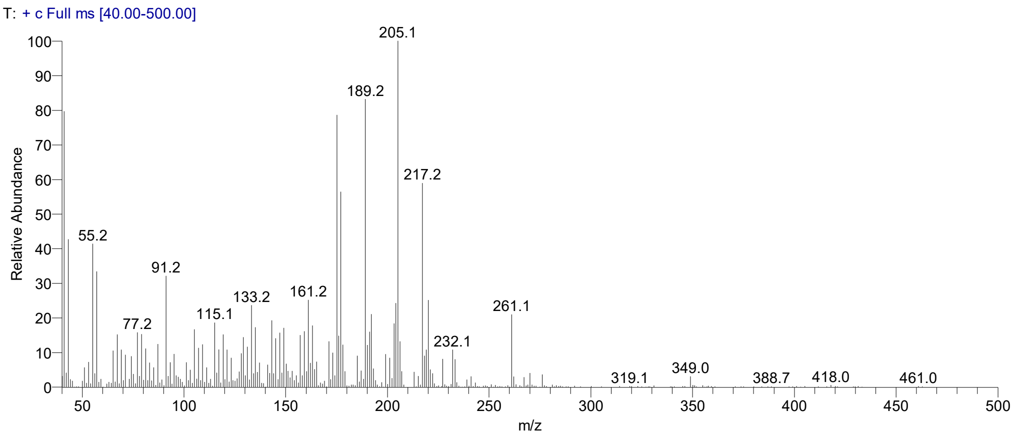
T: + c Full ms [40.00-500.00]
Relative Abundance
m/z
205.1
189.2
217.2
55.2
91.2
77.2
115.1
133.2
161.2
261.1
232.1
319.1
349.0
388.7
418.0
461.0

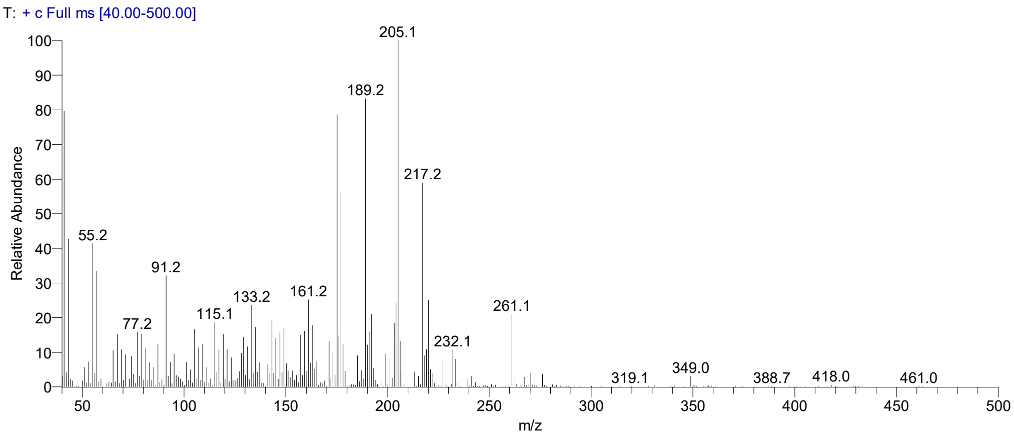
T: + c Full ms [40.00-500.00]
Relative Abundance
m/z
205.1
189.2
217.2
55.2
91.2
77.2
115.1
133.2
161.2
261.1
232.1
319.1
349.0
388.7
418.0
461.0

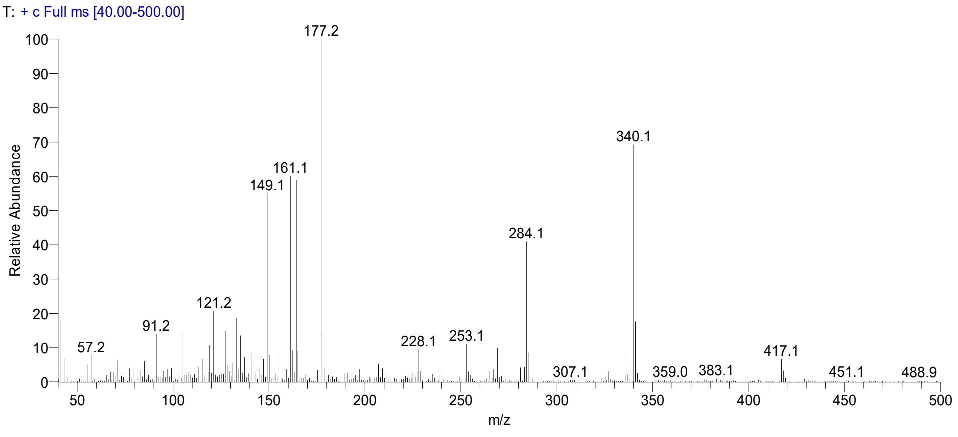
T: + c Full ms [40.00-500.00]
Relative Abundance
m/z
177.2
340.1
161.1
149.1
284.1
121.2
57.2
91.2
228.1
253.1
307.1
359.0
383.1
417.1
451.1
488.9

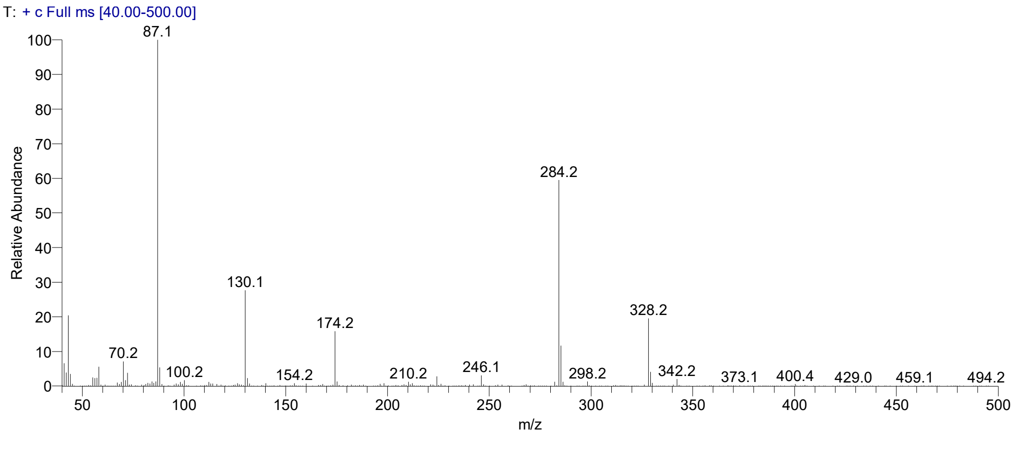
T: + c Full ms [40.00-500.00]
Relative Abundance
m/z
87.1
284.2
130.1
328.2
174.2
70.2
100.2
154.2
210.2
246.1
298.2
342.2
373.1
400.4
429.0
459.1
494.2

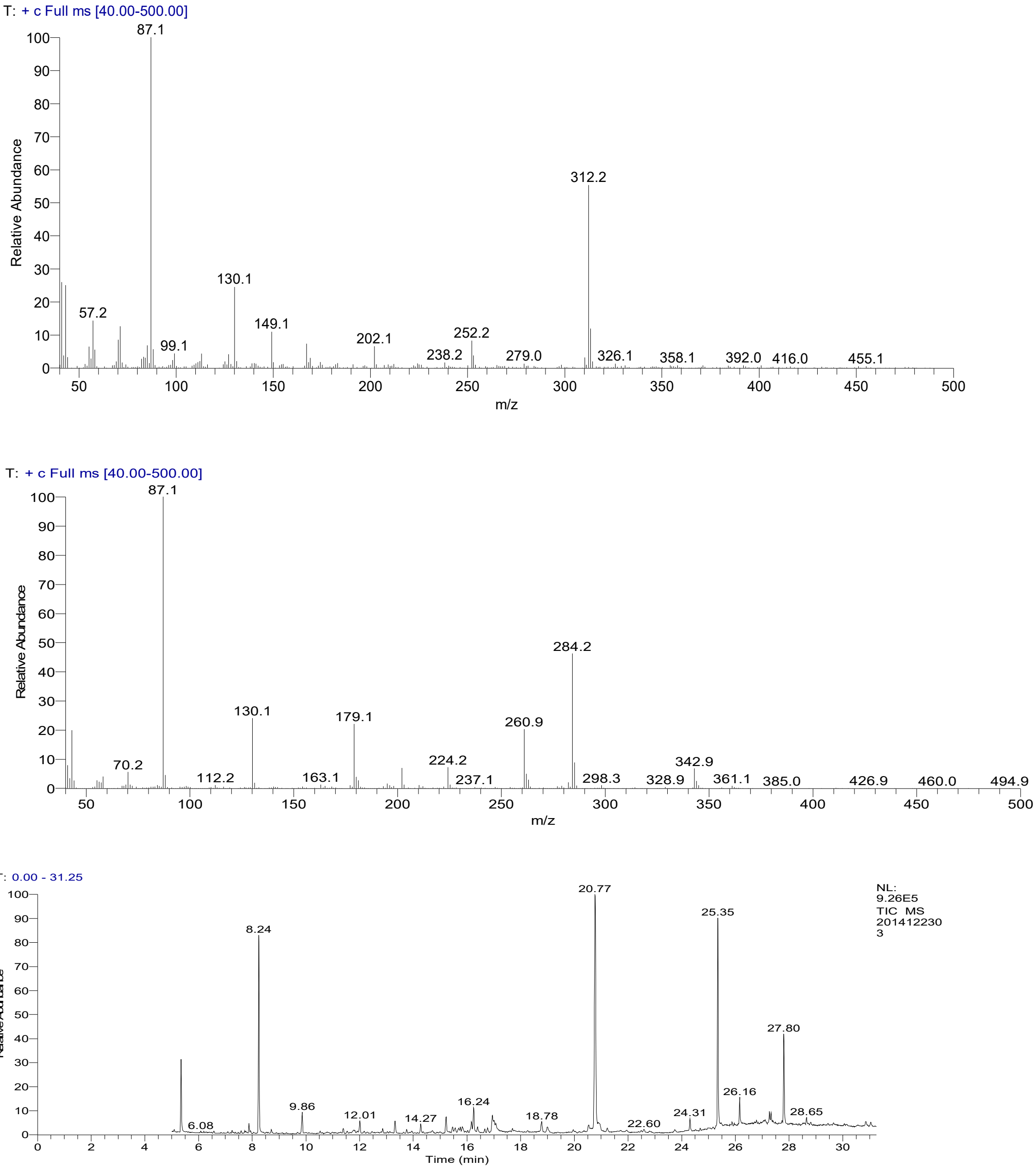


**Fig S3.** Total ion flow chromatogram of alditol acetate derivatives after fully methylated IPSW-3 hydrolysis reduction

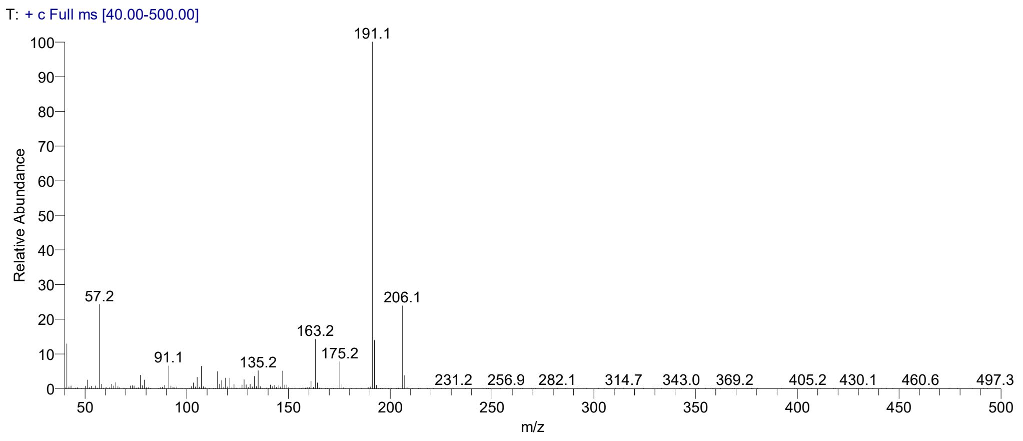
T: + c Full ms [40.00-500.00]
Relative Abundance
m/z
191.1
57.2
206.1
163.2
175.2
91.1
135.2
231.2
256.9
282.1
314.7
343.0
369.2
405.2
430.1
460.6
497.3

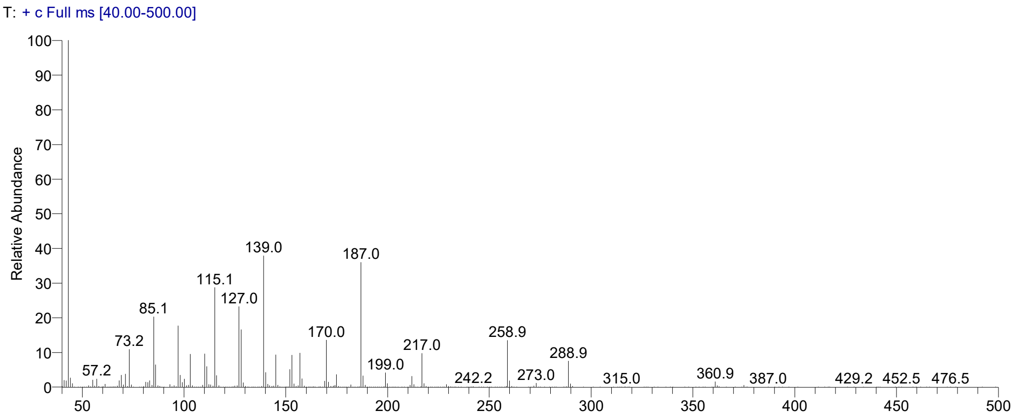
T: + c Full ms [40.00-500.00]
Relative Abundance
m/z
139.0
187.0
115.1
127.0
85.1
73.2
170.0
217.0
258.9
288.9
57.2
199.0
242.2
273.0
315.0
360.9
387.0
429.2
452.5
476.5

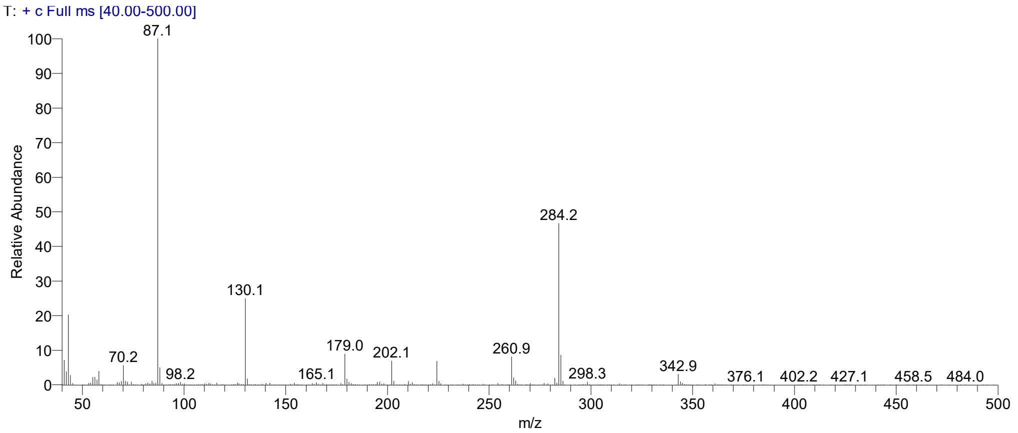
T: + c Full ms [40.00-500.00]
Relative Abundance
m/z
87.1
284.2
130.1
179.0
202.1
260.9
70.2
98.2
165.1
298.3
342.9
376.1
402.2
427.1
458.5
484.0

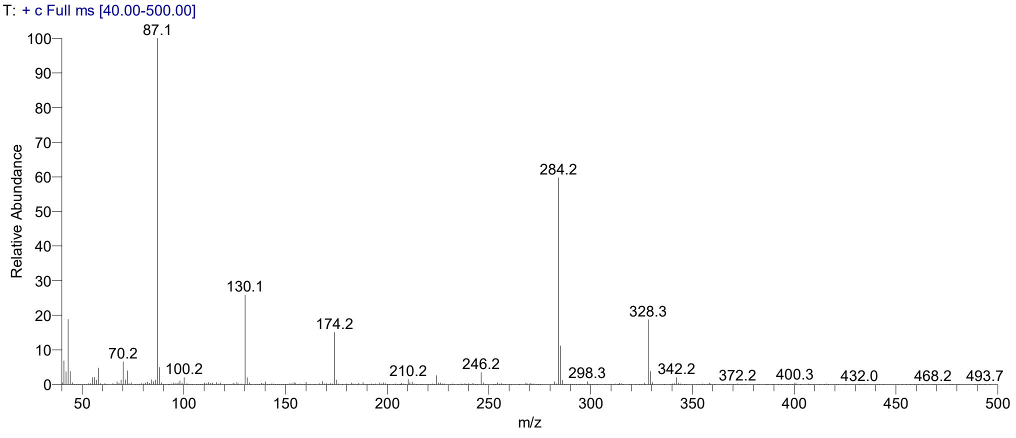
T: + c Full ms [40.00-500.00]
Relative Abundance
m/z
87.1
284.2
130.1
328.3
174.2
70.2
100.2
210.2
246.2
298.3
342.2
372.2
400.3
432.0
468.2
493.7

**Fig S4.** Total ion flow chromatogram of alditol acetate derivatives after fully methylated IPSW-4 hydrolysis reduction

**Table S1**. The results of periodate oxidation/Smith degration of different glycosyl linkage.

| Linkage | Consumption of periodate (mol/mol Glc) | Yield of formic acid (mol/mol Glc) | Retention time in GC of products of Smith degradation （min） | Products of Smith degradation |
|---|---|---|---|---|
| 1→2 | 1 | nd | / | Glycerol |
| 1→3 | nd | nd | / | Glucose/mannose |
| 1→4 | 1 | nd | / | Erythritol |
| 1→6 | 2 | 1 | / | Glycerol |
